# Rapid Infrarenal Aortic Rupture During Persistent Methicillin-Susceptible *Staphylococcus aureus* Bacteraemia: Serial CT Evolution and Staged Endovascular Treatment—A Case Report

**DOI:** 10.3390/diagnostics16213657

**Published:** 2026-11-05

**Authors:** Franciszek Kukliński, Michał Chlabicz, Arkadiusz Woźniak, Łukasz Rydzewski, Teresa Fernandez Moreno, Agnieszka Tycińska, Jerzy Głowiński

**Affiliations:** 1 Department of Vascular Surgery and Transplantation, Medical University of Bialystok, 15-276 Bialystok, Poland; michal.chlabicz@umb.edu.pl (M.C.); arekwozniak@o2.pl (A.W.); jglow@wp.pl (J.G.); 2 Department of Cardiology and Internal Medicine with Cardiac Intensive Care Unit, Medical University of Bialystok, 15-276 Bialystok, Poland; teresa.fernandez-moreno@sd.umb.edu.pl (T.F.M.); agnieszka.tycinska@gmail.com (A.T.)

**Keywords:** infective aortitis, methicillin-susceptible *Staphylococcus aureus*, aortic rupture, computed tomography angiography, endovascular aortic repair, type II endoleak, coil embolisation

## Abstract

**Background and Clinical Significance**: Persistent *Staphylococcus aureus* bacteraemia can seed an atherosclerotic aorta. Early CT may show only wall thickening and periaortic inflammation, mimicking a degenerative penetrating aortic ulcer (PAU) when no ulcer crater is visible. **Case Presentation**: An 80-year-old woman (BMI 19.5 kg/m^2^) presented with hypogastric and lumbar pain. Multiphasic CT showed a calcified infrarenal aorta with periaortic inflammation but no haemorrhage. Blood cultures obtained on two occasions confirmed persistent methicillin-susceptible *S. aureus* bacteraemia. Interval CT angiography showed an enhancing circumferential cuff and a small anterior contrast focus without a definite ulcer crater. Eight days after the initial CT, hypotension developed; CT angiography showed focal aortic wall disruption, active extravasation, and a large retroperitoneal haematoma. Emergency aorto-bi-iliac EVAR controlled the bleeding. An inferior mesenteric artery-fed type II endoleak filled the fresh rupture cavity and was treated by coil embolisation via mesenteric collaterals. One-month CTA showed a patent graft without endoleak. At six weeks, the patient remained clinically well with healed wounds and no need for further vascular intervention. CRP fell from 148.1 mg/L on postoperative day 5 to 49.4 mg/L at six weeks and 24.2 mg/L at approximately 12 weeks; the next outpatient review was scheduled in three months, although microbiological clearance remained undocumented. **Conclusions**: Serial imaging and persistent MSSA bacteraemia support probable infective aortitis with focal wall disruption; an infected degenerative PAU remains possible. Persistent bacteraemia, focal pain, and a progressive periaortic cuff should prompt early vascular review and short-interval imaging.

## 1. Introduction

A penetrating aortic ulcer (PAU) is an atherosclerotic plaque that disrupts the internal elastic lamina and extends into the media. On CT angiography, it usually appears as a focal contrast-filled crater or outpouching through a calcified intimal surface [1,2]. Aortic infection can look different at first. Early findings include asymmetric or circumferential wall thickening, periaortic fat stranding, and enhancing soft tissue. Later findings may include rapid focal enlargement, a saccular pseudoaneurysm, gas, psoas extension, vertebral destruction, or rupture [3,4,5].

Clinical context is therefore central. Persistent *S. aureus* bacteraemia and a new periaortic abnormality strongly suggest haematogenous aortic infection, but neither proves wall infection. The Academic Research Consortium for Infective Native Aortic Aneurysm (ARC of INAA) combines clinical, microbiological, and imaging evidence and also recognises the importance of tissue when it is available [6,7]. In emergency endovascular treatment, tissue is usually not obtained. A degenerative PAU complicated by infection, primary infective aortitis with focal wall disruption, and an infected pseudoaneurysm may then remain competing explanations.

This report addresses a practical diagnostic gap: how to interpret a progressive periaortic cuff in a small, heavily calcified infrarenal aorta when MSSA bacteraemia persists and no definite ulcer crater is visible. Serial CT captured the change from periaortic inflammation to active rupture over eight days. The case also shows the staged treatment of an early inferior mesenteric artery (IMA)-fed type II endoleak that communicated with the recent rupture cavity.

## 2. Materials and Methods

### 2.1. Clinical Data and Imaging Review

The clinical record, laboratory results, microbiology reports, procedure notes, and de-identified CT examinations were reviewed for this case report. The report follows the CARE guideline [8]. Hospital day 1 denotes admission to the referring hospital; postoperative day 0 denotes emergency endovascular aortic repair (EVAR). CT examinations were reviewed in axial planes and with multiplanar reconstructions. Aortic diameters were measured outer-to-outer on planes perpendicular to the vessel centreline where possible. The distinction among PAU, infective aortitis, and infective native aortic aneurysm or pseudoaneurysm was assessed from the combined clinical, microbiological, and imaging evidence. Image measurements not recorded prospectively are labelled as approximate retrospective estimates.

### 2.2. Use of Generative Artificial Intelligence

The manuscript preparation was supported by OpenAI ChatGPT (GPT-5 series; OpenAI, San Francisco, CA, USA; accessed 22 October 2026), which assisted with English translation, drafting and language editing based on author-supplied content, literature discovery, document formatting, and the preliminary technical assembly of de-identified CT panels and a procedural schematic based on the clinical records. The tool did not generate or alter source clinical data. The final image selection, measurements, phase labels, references, diagnostic interpretation, and scientific conclusions were independently checked, corrected where necessary, and approved by the authors, who take full responsibility for the work.

## 3. Case Presentation

### 3.1. Initial Presentation and Early Imaging

An 80-year-old woman (body mass index, 19.5 kg/m^2^) was admitted to a referring hospital with severe hypogastric pain radiating to the lumbar region and general weakness. The medical history included hypertension, type 2 diabetes, generalised atherosclerosis, asthma, chronic macrocytic anaemia, gout, and remote gastric cancer treated with total gastrectomy, splenectomy, omentectomy, and D2 lymphadenectomy. Coeliac and superior mesenteric artery stenoses had been treated by endovascular angioplasty during the preceding year. No prosthetic mesenteric bypass was present. Review of the available CT images showed no collection or gas around the treated mesenteric arteries.

On hospital day 2, the white blood cell count was 21.28 × 10^9^/L, haemoglobin was 10.6 g/dL, and creatinine was 1.25 mg/dL. Multiphasic CT showed a heavily calcified infrarenal aorta with wall thickening and ill-defined periaortic fat stranding. There was no retroperitoneal haematoma, active extravasation, early saccular contour, or definite contrast-filled ulcer crater on retrospective multiplanar review. No periaortic gas, psoas collection, vertebral destruction or spondylodiscitis, or direct aortoenteric communication was present. The fat plane between the aorta and duodenum was preserved.

Ultrasonography on hospital day 3 described a 30 × 27 mm “aortic contour”, a 12 mm anterior mural component, a 56 × 12 × 63 mm hypoechoic periaortic region, and a possible 2 mm anterior wall discontinuity. The report did not state whether the measurement was inner-to-inner or outer-to-outer or whether it was obtained perpendicular to the aortic axis. Retrospective orthogonal outer-to-outer CT measurement showed a maximal native diameter of approximately 21 mm at the lesion and 18–19 mm in adjacent normal-appearing segments, a ratio of about 1.1–1.2. The ultrasound value reflected inclusion of thickened wall or periaortic tissue and use of a non-orthogonal contour. The CT dimensions were below both the conventional 30 mm abdominal aneurysm threshold and the ≥1.5-fold relative-diameter criterion, which is relevant in a small patient.

**Figure 1:**
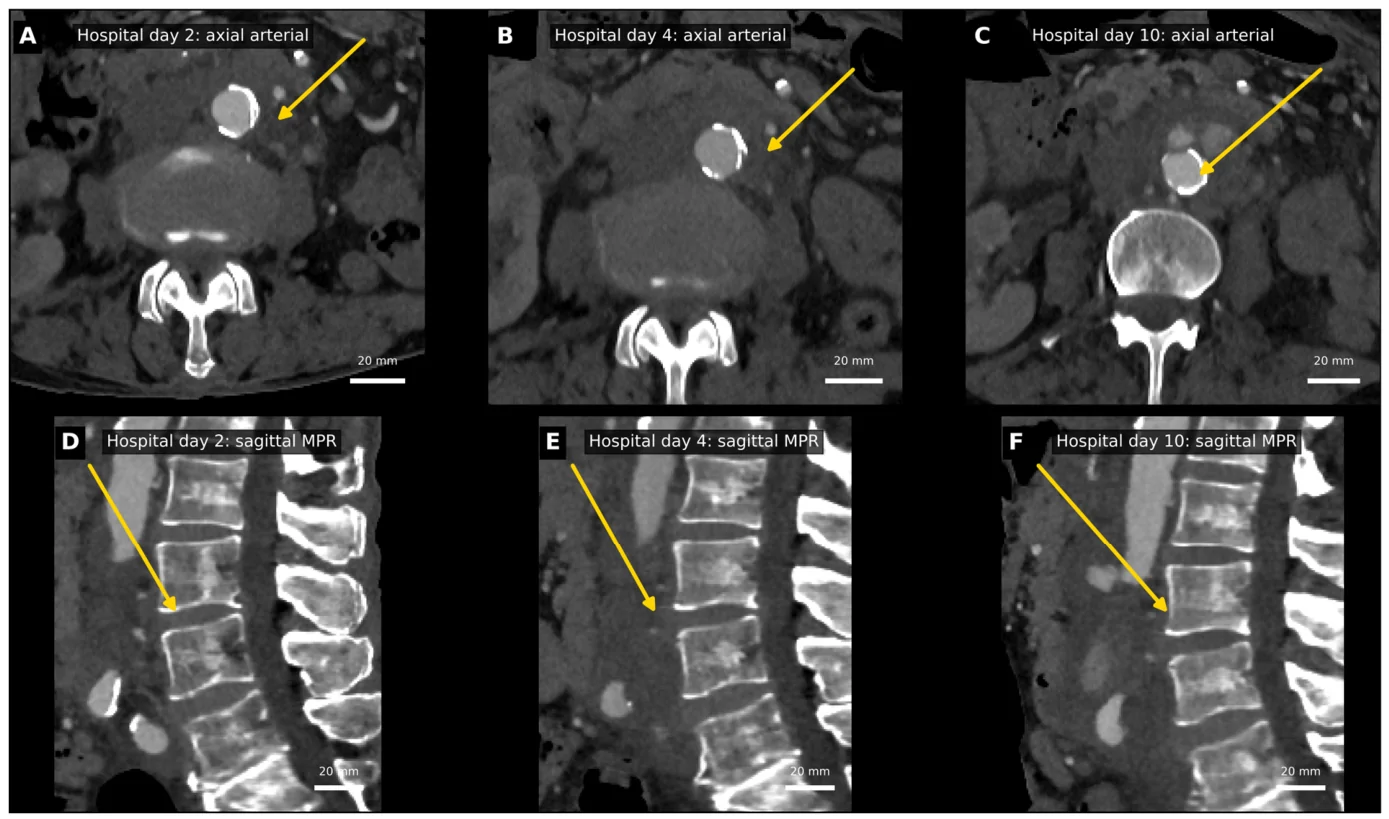
Serial CT evolution at matched infrarenal levels. (**A**,**D**) Hospital-day-2 arterial-phase axial image and sagittal multiplanar reconstruction (MPR) show ill-defined wall and periaortic inflammatory change around a heavily calcified, small aorta. (**B**,**E**) Hospital-day-4 CTA shows a thicker, enhancing circumferential cuff and focal anterior contour abnormality near the expected inferior mesenteric artery origin. No definite contrast-filled ulcer crater is seen. (**C**,**F**) Hospital-day-10 CTA shows focal wall disruption with active contrast extravasation into a new retroperitoneal haematoma. Yellow arrows mark the evolving abnormality. Images were selected at corresponding anatomical levels; scale bars, 20 mm.

CT angiography (CTA) on hospital day 4 showed a more sharply defined, circumferentially enhancing periaortic cuff up to 14 mm thick, extending caudally from near the renal arteries. There was no haemorrhage or extravasation. An approximately 8.8 mm contrast-containing focus lay anterior to the aorta near the expected IMA origin, which was not separately seen. This finding could represent a small pseudoaneurysmal contour change or an enhancing vascular channel; it did not have a clearly demonstrable neck or crater penetrating an atherosclerotic plaque. Figure 1 shows the change at matched infrarenal levels and on sagittal reconstructions.

The hospital-day-4 study was initially interpreted as an inflammatory periaortic process without active bleeding. Because no definite ulcer crater, pseudoaneurysm, or haematoma was present and the patient was receiving antibiotics, conservative treatment was continued. The available record does not document a formal vascular surgery consultation, consideration of prophylactic repair, or a prespecified short-interval repeat CTA before clinical deterioration. In retrospect, the combination of pain, persistent bacteraemia, progressive enhancement, and a focal contour change warranted early vascular review and closer imaging surveillance.

### 3.2. Microbiology and Antimicrobial Treatment

A blood multiplex polymerase chain reaction test on hospital day 3 detected *S. aureus* without a resistance marker. Aerobic and anaerobic blood cultures collected on hospital day 4 grew MSSA (oxacillin minimum inhibitory concentration ≤0.25 mg/L). Repeat aerobic and anaerobic cultures collected on hospital day 8 again grew MSSA despite antimicrobial treatment. C-reactive protein (CRP) peaked at 322 mg/L and the white blood cell count at 25.50 × 10^9^/L. Meropenem at 1 g intravenously three times daily (TID) was started on hospital day 3. Cloxacillin at 2 g intravenously every 6 h was added on hospital day 4 after MSSA identification. The documented inpatient courses lasted approximately 18 and 17 days, respectively, through discharge. The record did not explain why meropenem was continued after susceptibility results became available and did not contain a formal infectious diseases consultation.

Right-parotid inflammation was present without a drainable abscess. It could have been the portal of entry or a metastatic focus, but the source remained uncertain. Transthoracic echocardiography showed a left-ventricular ejection fraction of 65% and no visible vegetation. Transoesophageal echocardiography was not performed. No follow-up blood culture documenting clearance was available.

### 3.3. Rupture and Emergency Endovascular Repair

**Figure 2:**
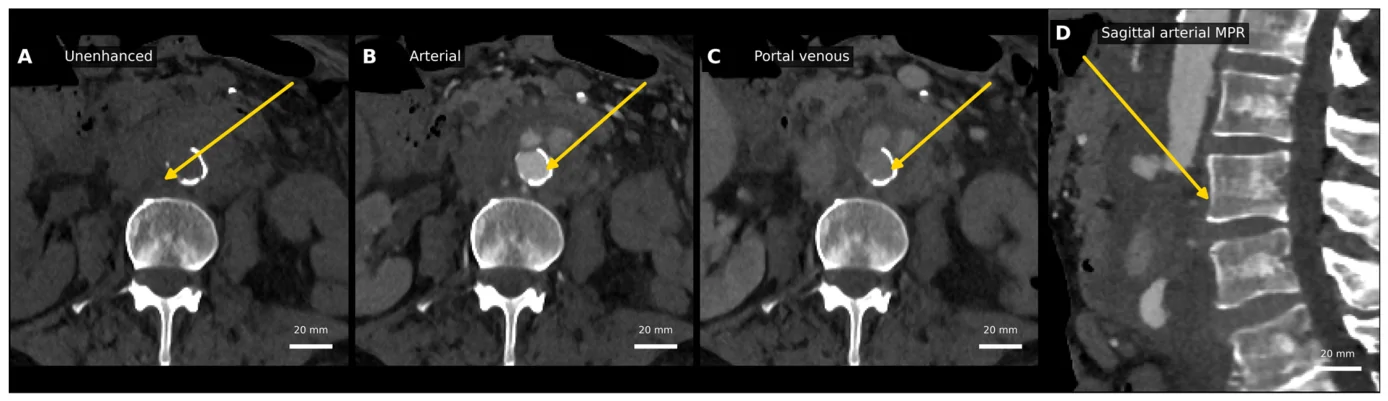
Multiphasic CT on hospital day 10. (**A**) The unenhanced image shows a new retroperitoneal haematoma. (**B**) The arterial phase shows a focal contrast jet contiguous with the anterior aortic lumen. (**C**) Contrast persists in the portal venous phase. (**D**) Sagittal arterial MPR shows the focal wall disruption and adjacent haematoma. Yellow arrows mark the rupture site; scale bars, 20 mm.

On hospital day 10, eight days after the initial CT, ultrasound showed enlargement of the lower aortic and periaortic complex to 47 mm and a new 28 × 42 mm adjacent haematoma. CTA demonstrated focal anterior aortic wall disruption near the IMA level, active contrast extravasation, and a 70 × 40 × 100 mm retroperitoneal haematoma extending from below the renal arteries to the iliac level (Figure 2). The native aorta remained approximately 21 mm in diameter. Blood pressure fell to 78/41 mmHg before transfer. At the vascular centre, temperature was 36.6 °C, blood pressure was 106/69 mmHg, and heart rate was 52 beats/min. The abdomen was soft and non-tender, and limb perfusion was preserved. Haemoglobin was 9.9 g/dL, white blood cell count was 16.67 × 10^9^/L, creatinine was 0.95 mg/dL, and fibrinogen was 746 mg/dL.

The emergency diagnosis was focal rupture of a small infrarenal aorta during persistent MSSA bacteraemia. The overall pattern was most consistent with probable infective aortitis with focal wall disruption. A degenerative PAU complicated by infection and an infected pseudoaneurysm remained differential diagnoses because no aortic tissue was obtained and no definite pre-rupture ulcer crater had been demonstrated.

Retrospective review of the rupture CTA estimated approximately 43 mm from the lowest renal artery to the lesion, an infrarenal aortic diameter of 19–21 mm, neck angulation below 30°, an aortic bifurcation calibre of about 15 mm, common iliac diameters of 8–9 mm, distal iliac sealing lengths greater than 25 mm, and minimum iliofemoral access diameters of about 6 mm. These measurements supported endovascular access and infrarenal sealing. They should be regarded as retrospective estimates rather than a formal device-planning worksheet.

Emergency EVAR was performed under general anaesthesia through bilateral percutaneous femoral access. The operative note did not record a formal comparison with local anaesthesia, but recent hypotension, active bleeding, and the possible need for a complex multicomponent repair supported airway and haemodynamic control under general anaesthesia. The note did not identify the percutaneous closure devices; it also did not record any access difficulty or access-related complication.

**Figure 3:**
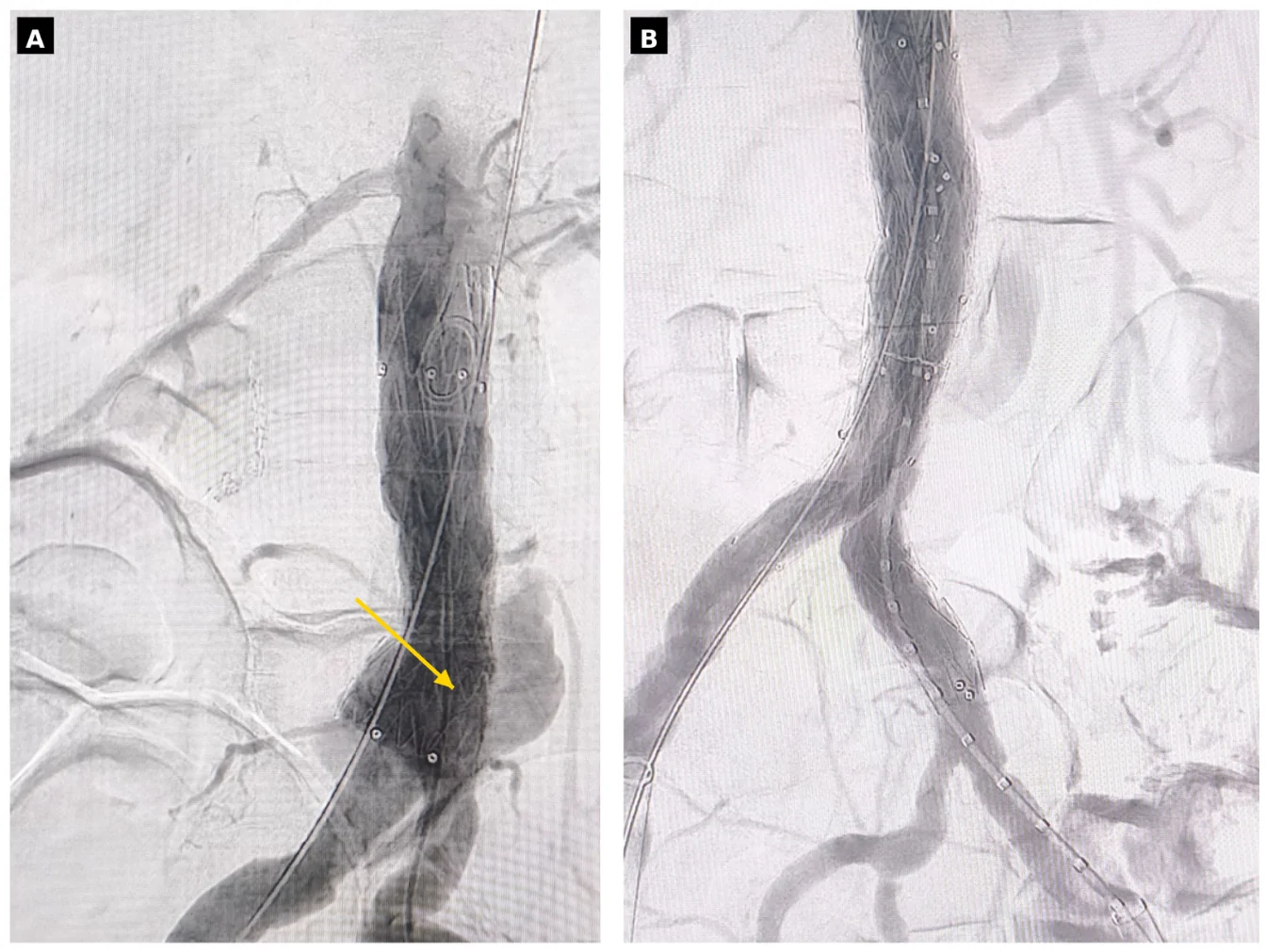
Intraoperative aortography during emergency EVAR. (**A**) Persistent contrast extravasation after deployment of the initially available Medtronic Endurant II abdominal tube graft (ETTF2323C70EE), which did not cover the full distal extent of the diseased segment (arrow). (**B**) Final aortography after placement of the modular Endurant II/IIs aorto-bi-iliac configuration shows complete exclusion of the rupture and no residual arterial-phase extravasation. EVAR, endovascular aortic repair.

Aortography showed high-volume extravasation near the IMA level. Because of the immediately life-threatening haemorrhage and recent hypotension, the operating team first deployed a Medtronic Endurant II abdominal tube graft (Medtronic Vascular, Santa Rosa, CA, USA; ETTF2323C70EE; 23/23 mm diameter, 70 mm covered length), which was immediately available for rapid damage control. Aortography showed persistent extravasation because this component did not cover the full distal extent of the diseased segment (Figure 3A). The repair was therefore extended during the same procedure with a modular Medtronic Endurant II/IIs aorto-bi-iliac configuration comprising an Endurant IIs bifurcated body (ESBF2314C103EE; 23/14 mm proximal/distal diameters; 103 mm covered length), a right Endurant II iliac limb extension (ETLW1616C82EE; 16/16 mm proximal/distal diameters; 82 mm covered length), and a left Endurant II iliac limb extension (ETLW1616C124EE; 16/16 mm proximal/distal diameters; 124 mm covered length). The covered proximal sealing zone was infrarenal, immediately below the left renal artery, while the uncovered fixation stent extended suprarenally; both limbs ended in the common iliac arteries. Final aortography confirmed complete exclusion of the rupture and no residual arterial-phase extravasation (Figure 3B). Delayed IMA opacification was suspected, but a type II endoleak was not yet proven. With haemorrhage controlled, we deferred additional IMA catheterisation in favour of early postoperative CTA. The operative records did not provide exact oversizing or component overlap, confirmation of instructions-for-use compliance, procedure or fluoroscopy times, radiation dose metrics, or contrast volume.

### 3.4. Early Type II Endoleak and Coil Embolisation

**Figure 4:**
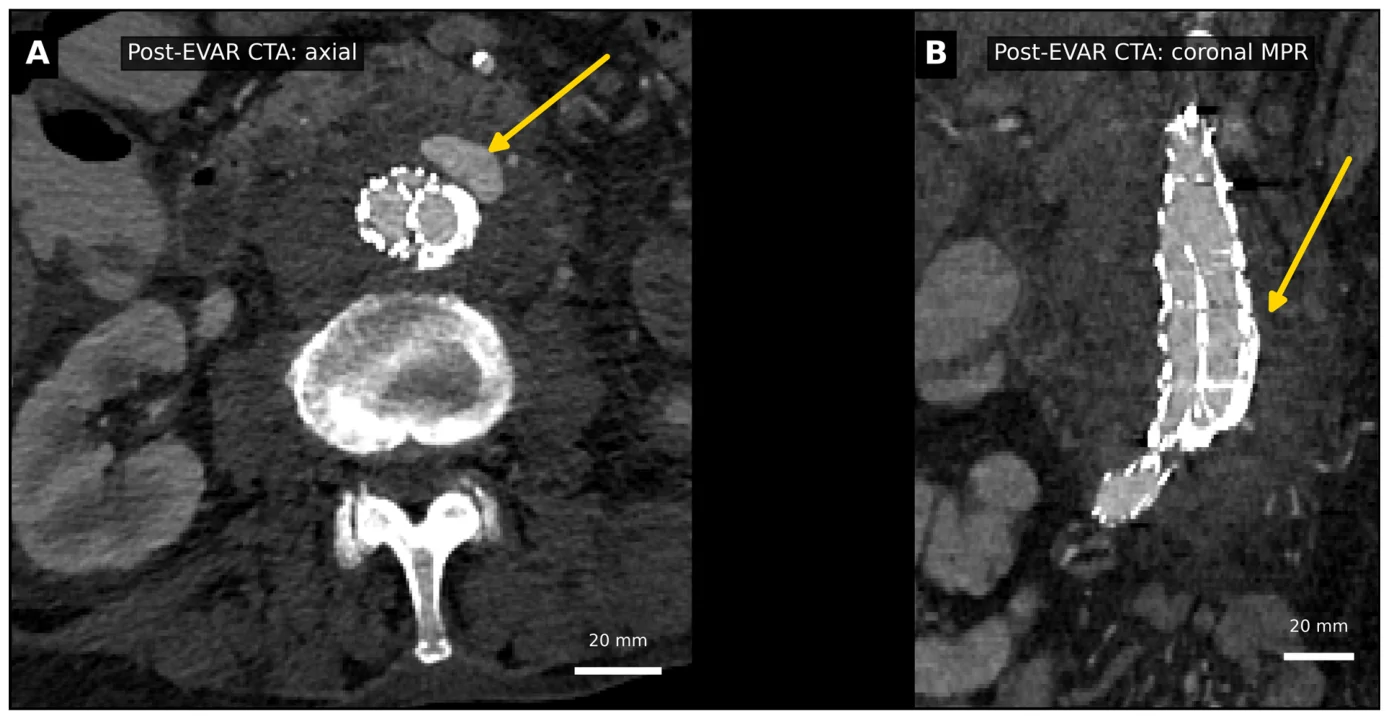
CTA on postoperative day 2 after EVAR. (**A**) Axial contrast-enhanced image and (**B**) coronal MPR show focal contrast opacification within the recent rupture cavity adjacent to the endograft (yellow arrows), consistent with an IMA-fed type II endoleak. No type Ia or Ib endoleak was present. Scale bars, 20 mm.

CTA on postoperative day 2 showed a patent aorto-bi-iliac endograft with an infrarenal covered sealing zone and suprarenal bare-stent fixation. Both renal arteries and the superior mesenteric artery were patent, with no type Ia or Ib endoleak. A type II endoleak supplied by the IMA opacified the recently ruptured cavity beside the graft (Figure 4). This was not an incidental low-flow endoleak in a stable aneurysm sac: the perfused space was the fresh rupture cavity. Continued retrograde pressurisation was therefore considered unsafe, and embolisation was planned.

The patient remained haemodynamically stable while the intervention was arranged. Embolisation was performed on postoperative day 6, four days after CTA diagnosis. The record does not state another reason for this interval. From left-brachial access, the IMA inflow was reached through superior mesenteric-to-IMA collaterals and occluded with coils. The coil brand and number were not recorded in the available note. Completion angiography showed no further endoleak filling. Selective angiographic frames from the later embolisation were not available in the supplied archive; the SMA-to-IMA collateral route and coil deployment are therefore described from the procedure report rather than shown in Figure 3.

### 3.5. Clinical Outcome and Follow-Up

The subsequent inpatient course was haemodynamically stable and afebrile. Serial abdominal examinations were benign, and limb perfusion remained normal. Two units of packed red blood cells (PRBCs) were transfused without reaction on postoperative day 2 when haemoglobin was 8.3 g/dL; two further PRBC units were given on postoperative day 8 when haemoglobin reached 7.4 g/dL. CRP fell from 148.1 mg/L on postoperative day 5 to 102 mg/L on day 6 and 58.6 mg/L on day 8, and procalcitonin was 0.11 ng/mL. At discharge on postoperative day 9, the patient had no abdominal pain; white blood cell count was 9.50 × 10^9^/L, haemoglobin was 9.7 g/dL, and creatinine was 0.79 mg/dL.

**Figure 5:**
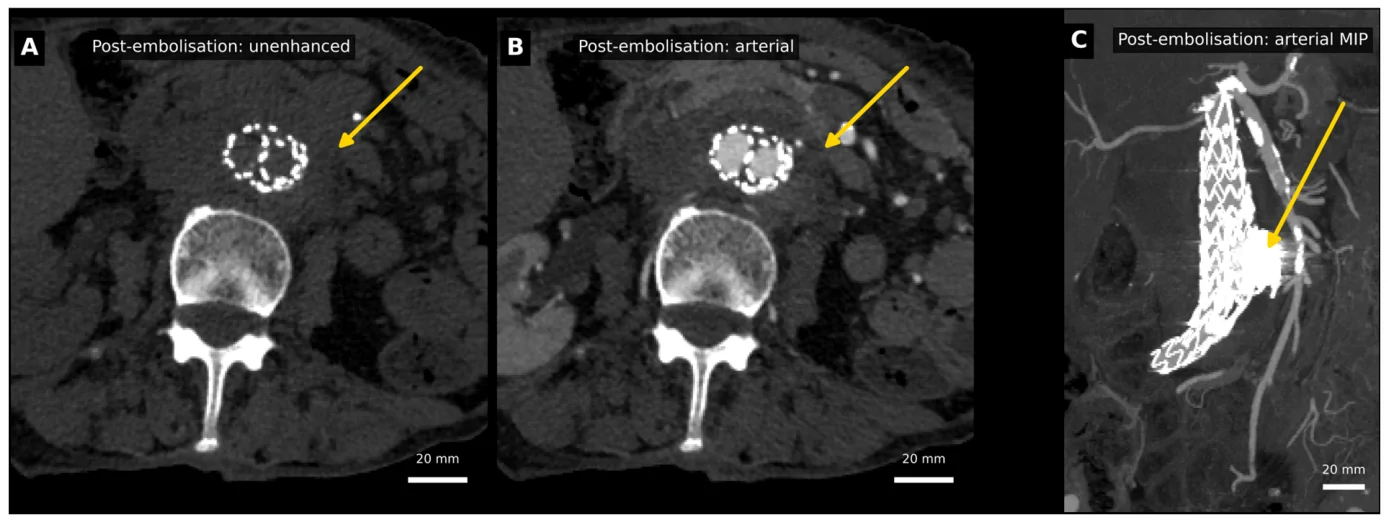
CTA 23 days after coil embolisation. Matched (**A**) unenhanced and (**B**) arterial-phase axial images show the endograft and excluded rupture cavity without contrast filling; coil material is visible as adjacent high-attenuation foci. (**C**) Coronal arterial maximum-intensity projection shows patent endograft flow and no residual endoleak. In panels (**A**,**B**), the arrows mark the excluded rupture cavity; in panel (**C**), the arrow indicates the coil pack. Scale bars, 20 mm.

CTA 23 days after embolisation and 29 days after EVAR showed a patent aorto-bi-iliac endograft, coil material in place, and no residual endoleak (Figure 5). This demonstrated early anatomical success but did not establish microbiological cure.

At five-week telephone follow-up, the patient reported no recurrent abdominal or back pain, wound problem, or rehospitalisation. A brief episode of fever and raised blood pressure soon after discharge had resolved. The patient reported taking levofloxacin 500 mg once daily for 10 days after discharge. No prescribing record or rationale for this oral step-down was available. At approximately six weeks, vascular review found healed groin wounds and no indication for further vascular intervention. CRP had fallen from 148.1 mg/L on postoperative day 5 to 102 mg/L on day 6 and 58.6 mg/L on day 8; it remained above normal at 49.4 mg/L at the six-week review. The white blood cell count was 11.69 × 10^9^/L, haemoglobin was 9.7 g/dL, creatinine was 1.26 mg/dL, and estimated glomerular filtration rate was 43 mL/min/1.73 m^2^. No further antimicrobial treatment was documented at this clinically stable vascular review. At approximately 12 weeks after EVAR, CRP had decreased further to 24.2 mg/L. The next outpatient review was scheduled for three months later. No follow-up blood culture result was available. Thus, clinical, anatomical, and biochemical improvement was documented, while microbiological clearance remained unverified.

The clinical, microbiological, imaging, and treatment course is summarised in Table 1.

## 4. Discussion

### 4.1. Diagnostic Interpretation

Infective aortitis is uncommon but can cause rapid aortic wall destruction, pseudoaneurysm formation, and rupture. Because early imaging findings may be subtle, diagnosis requires integration of serial CT findings, blood culture results, and clinical progression [3,4,5,9].

The serial CT pattern and persistent MSSA bacteraemia make infective aortitis with focal wall disruption the leading diagnosis. The first CT showed periaortic inflammation without an aneurysm. The next study showed circumferential enhancement and a small anterior contrast-containing focus. Eight days after the first CT, the same region ruptured. This sequence resembles the early oedema and rapid progression described in infected aortic lesions [3,4,5]. Absence of gas, a mature saccular pseudoaneurysm, psoas infection, or vertebral destruction does not exclude infection; these findings are variably present and may occur late [3,4,5].

The original diagnosis of abdominal PAU was understandable because the aorta was heavily calcified and the disruption arose near an atherosclerotic plaque. However, a PAU requires a focal crater extending through the intima into the media [1,2]. No definite crater was visible before rupture, even on retrospective sagittal and coronal review. Conversely, persistent MSSA bacteraemia, a progressive enhancing cuff, and rapid wall failure favour infection. A degenerative PAU secondarily infected by MSSA cannot be excluded, and the focal day-4 contrast focus might have represented an early infected pseudoaneurysmal change. No tissue was obtained, so the case should not be labelled as histologically proven infective aortitis or as a proven PAU.

The main imaging and clinical differential diagnoses are summarised in Table 2.

Available published cases show that *S. aureus* aortitis can progress rapidly to rupture [9]. A recent report from our centre described a small, clearly visible infrarenal PAU that developed contained rupture after three weeks of MSSA bacteraemia [10]. The present case differs because no pre-rupture ulcer crater was shown, the first abnormality was mainly periaortic, and free extravasation occurred within eight days. These differences support interpreting this as primary infection-associated wall injury rather than a purely degenerative PAU.

### 4.2. Timing of Vascular Assessment

The most important early warning signs were not aortic size but change over time: persistent focal pain, repeat MSSA-positive cultures, increasing periaortic enhancement, and a new focal contour abnormality. The day-4 CTA showed no bleeding and no definite repair target, which explains initial caution. Even so, the combination should have triggered urgent vascular and infectious diseases assessment and a defined short-interval CTA plan. Waiting for a classic saccular pseudoaneurysm or gas risks missing the short window before rupture. This is the principal clinical lesson of the case.

### 4.3. Emergency EVAR in a Potentially Infected Field

EVAR achieved immediate haemorrhage control in an 80-year-old patient with recent hypotension and active extravasation. Open debridement and reconstruction would provide tissue and remove the infected wall, but it carries major early physiological stress. A meta-analysis of infective native aortic aneurysms reported lower pooled 30-day mortality after EVAR than open repair (4.9% versus 11.7%) but more infection-related complications during follow-up (23.2% versus 8.2%) [11]. These observational data are heterogeneous and do not define the best treatment for every patient, but they explain why EVAR can be a life-saving first strategy while leaving an unresolved infection risk. In this case, the intraoperative sequence reflected the emergency setting and immediate device availability rather than a planned elective configuration. The straight component was used first in an attempt to obtain the fastest possible damage control. Persistent extravasation prompted same-procedure extension with an overlapping bifurcated endograft, after which the rupture was completely excluded (Figure 3).

The 2026 ESVS guideline concerns vascular graft and endograft infection, not primary native-aortic infection [12]. It becomes relevant here because a prosthetic endograft was placed across a probably infected native segment. Clinical recovery and a normal one-month CTA are reassuring, but they do not exclude later endograft infection. Therefore, long-term clinical, inflammatory-marker, microbiological, and imaging surveillance is needed; recurrent pain, fever, bacteraemia, periaortic gas or fluid, pseudoaneurysm, or graft-related change should prompt renewed investigation [7,12].

### 4.4. Antimicrobial Management

For MSSA bacteraemia, an antistaphylococcal beta-lactam such as cloxacillin or cefazolin is preferred once susceptibility is known [13]. Cloxacillin was appropriate, but the reason for continuing meropenem was not documented. Current recommendations call for repeat blood cultures every 24–48 h until clearance and support transoesophageal echocardiography when cultures remain positive for at least 48 h despite a negative transthoracic study [13,14]. Neither clearance cultures nor transoesophageal echocardiography were documented in this patient.

Persistent bacteraemia, a probable endovascular focus, and new prosthetic material define a complicated infection. A prolonged, pathogen-directed plan with infectious diseases input and follow-up would normally be expected [7,12,13,14]. The approximately 17-day inpatient cloxacillin course followed by a patient-reported 10-day course of oral levofloxacin, without documented clearance or a defined suppressive strategy, cannot establish eradication. The progressive decline in CRP to 24.2 mg/L at approximately 12 weeks was reassuring, although the value remained above normal and microbiological clearance had not been documented. Continued surveillance remains necessary, and early anatomical success should be reported separately from microbiological outcome.

### 4.5. Early IMA-Fed Type II Endoleak

Most type II endoleaks after elective EVAR are observed unless they persist with sac enlargement [15,16]. This case was different. The endoleak filled a fresh rupture cavity two days after emergency repair, so even modest retrograde pressure could threaten haemostasis. Transcollateral coil embolisation removed the documented IMA inflow, and one-month CTA showed no residual filling. The result supports early treatment in this specific anatomy but should not be extrapolated to an uncomplicated type II endoleak after elective AAA repair.

IMA embolisation was not performed during the index procedure because completion angiography showed no arterial-phase extravasation and only suspected delayed IMA opacification. In an unstable emergency, rapid exclusion took priority over unproven collateral filling. Once CTA confirmed communication with the rupture cavity, staged embolisation was justified. Continued imaging remains important because coil occlusion of a feeder does not exclude later recruitment of another collateral channel [15].

### 4.6. Considerations in Women

Women undergoing EVAR for abdominal aortic aneurysm often have smaller access arteries and more challenging proximal neck anatomy, which may increase technical difficulty and perioperative morbidity. Contemporary studies nevertheless report heterogeneous long-term results, including comparable five-year outcomes in selected women [17,18]. These data concern aneurysmal disease and cannot be directly transferred to an infected, small aortic lesion. They also relate mainly to access, proximal sealing, and type Ia endoleak, not the IMA-fed type II endoleak seen here. In this patient, small vessels were clinically relevant to planning, but bilateral percutaneous access and infrarenal sealing were achieved without a recorded access complication.

### 4.7. Limitations

This is a single case with short follow-up. No aortic tissue, transoesophageal echocardiogram, clearance blood culture, or documented infectious diseases treatment plan was available. Several device and radiation details and selective DSA frames from the later embolisation were absent from the records. Retrospective CTA measurements therefore cannot replace the original device worksheet. These limits prevent proof of the exact lesion origin and of microbiological cure, but they do not change the observed sequence of persistent MSSA bacteraemia, progressive periaortic inflammation, focal rupture, and early anatomical success after staged endovascular treatment.

## 5. Conclusions

Persistent MSSA bacteraemia, focal abdominal or back pain, and a progressive enhancing periaortic cuff should prompt early vascular consultation and short-interval aortic imaging, even when the aorta is small and no classic pseudoaneurysm is present. In this patient, serial CT documented progression to rupture within eight days. Emergency EVAR controlled haemorrhage, and targeted coil embolisation treated an IMA-fed type II endoleak that filled the recent rupture cavity. The short-term anatomical result was favourable, but long-term infection and graft surveillance remain essential because microbiological clearance was not documented.

## 6. Previous Presentations

The authors presented a Polish-language abstract and poster based on this case at the 2026 Conference of the Polish Cardiac Society Section of Heart Diseases in Women, Kraków, Poland, on 10 October 2026. The poster presentation received first prize at the conference. Preliminary findings from this case were also presented orally as “Ruptured Abdominal Aortic Lesion in a Patient With Staphylococcus aureus Bacteremia: A Challenging Endovascular Case” at the Warsaw International Vascular Meeting (WIVAM 2026), Warsaw, Poland, on 17 October 2026, in International Scientific Session 1. The oral presentation received first prize in that session. Neither conference presentation constituted a prior publication of the full manuscript.

## Figures and Tables

**Table 1:** Clinical, microbiological, imaging, and treatment timeline.

Time	Key Findings	Management or Outcome
Day 1	Severe hypogastric and lumbar pain; BMI 19.5 kg/m^2^.	Admission to referring hospital.
Day 2	WBC 21.28 × 10^9^/L. Multiphasic CT: small calcified infrarenal aorta, wall thickening and periaortic stranding; no crater, haematoma, or extravasation.	Clinical and laboratory surveillance.
Days 3 and 4	Blood PCR and cultures: MSSA. Ultrasound reported a 30 × 27 mm contour. CTA: enhancing cuff up to 14 mm and an 8.8 mm anterior contrast focus; no bleeding.	Meropenem started, then cloxacillin added. Conservative management continued.
Day 5	TTE: no vegetation. Parotid inflammation without abscess.	Source of bacteraemia remained uncertain; no TEE documented.
Day 8	Repeat aerobic and anaerobic blood cultures again grew MSSA.	Persistent bacteraemia established.
Day 10	Eight days after initial CT: hypotension, focal aortic disruption, active extravasation, and 70 × 40 × 100 mm retroperitoneal haematoma.	Urgent transfer and emergency EVAR.
POD 2	CTA: IMA-fed type II endoleak filling the fresh rupture cavity; no type I endoleak.	Two red-cell units; embolisation planned.
POD 6	Collateral route from SMA to IMA catheterised.	Coil embolisation; no residual filling on completion angiography.
POD 5–9	CRP 148.1, 102, and 58.6 mg/L on POD 5, 6, and 8, respectively; haemoglobin nadir 7.4 g/dL; discharge WBC 9.50 × 10^9^/L, haemoglobin 9.7 g/dL, creatinine 0.79 mg/dL.	Two further red-cell units; discharge without abdominal pain.
Day 29 after EVAR	CTA: patent graft, coils in place, no endoleak.	Early anatomical success.
Six-week review	Clinically well, wounds healed; CRP 49.4 mg/L, WBC 11.69 × 10^9^/L, haemoglobin 9.7 g/dL, creatinine 1.26 mg/dL.	Clinically stable with healed wounds; no further vascular intervention; outpatient surveillance continued.
Approximately 12 weeks after EVAR	CRP 24.2 mg/L.	Continued outpatient surveillance; next review scheduled in 3 months.

BMI, body mass index; CRP, C-reactive protein; CTA, computed tomography angiography; EVAR, endovascular aortic repair; IMA, inferior mesenteric artery; MSSA, methicillin-susceptible *Staphylococcus aureus*; PCR, polymerase chain reaction; POD, postoperative day; SMA, superior mesenteric artery; TEE, transoesophageal echocardiography; TTE, transthoracic echocardiography; WBC, white blood cell count.

**Table 2:** Imaging and clinical differential diagnosis.

Diagnosis	Supporting Features	Features Against or Unresolved
Degenerative PAU with secondary infection	Heavy aortic calcification; focal rupture beside an atherosclerotic plaque.	No definite pre-rupture contrast-filled crater; circumferential inflammatory cuff and persistent MSSA bacteraemia were dominant.
Primary infective aortitis with focal wall disruption	Two MSSA-positive culture episodes; severe inflammation; progressive enhancing periaortic tissue; rapid rupture at the same level.	No wall culture or histology; no periaortic gas.
Infected pseudoaneurysm/INAA	Small anterior contrast focus on day 4 and later focal rupture cavity.	No established saccular aneurysm before rupture; CT diameter was only about 1.1–1.2 times adjacent aorta.
Non-infectious inflammatory aortitis or retroperitoneal fibrosis	Circumferential periaortic soft tissue.	Persistent MSSA bacteraemia, focal wall disruption, and rapid haemorrhagic progression argue against it.
Aortoenteric fistula	Prior abdominal surgery and aortic inflammation could raise concern.	No gastrointestinal bleeding, periaortic gas, or loss of the duodenal fat plane; no direct communication on CT.

INAA, infective native aortic aneurysm; MSSA, methicillin-susceptible *Staphylococcus aureus*; PAU, penetrating aortic ulcer.

## Data Availability

The data supporting this case report are contained within the article. Further anonymised clinical and imaging data are available from the corresponding author on reasonable request, subject to institutional approval and patient confidentiality.
